# Lollipops in the Clinic: Information Dense Mutation Plots for Precision Medicine

**DOI:** 10.1371/journal.pone.0160519

**Published:** 2016-08-04

**Authors:** Jeremy J. Jay, Cory Brouwer

**Affiliations:** 1 Bioinformatics Services Division, North Carolina Research Campus, Kannapolis, North Carolina, United States of America; 2 Bioinformatics and Genomics Department, University of North Carolina at Charlotte, Charlotte, North Carolina, United States of America; National Cancer Institute, UNITED STATES

## Abstract

**Introduction:**

Concise visualization is critical to present large amounts of information in a minimal space that can be interpreted quickly. Clinical applications in precision medicine present an important use case due to the time dependent nature of the interpretations, although visualization is increasingly necessary across the life sciences. In this paper we describe the Lollipops software for the presentation of panel or exome sequencing results. Source code and binaries are freely available at https://github.com/pbnjay/lollipops. Although other software and web resources exist to produce lollipop diagrams, these packages are less suited to clinical applications. The demands of precision medicine require the ability to easily fit into a workflow and incorporate external information without manual intervention.

**Results:**

The Lollipops software provides a simple command line interface that only requires an official gene symbol and mutation list making it easily scriptable. External information is integrated using the publicly available Uniprot and Pfam resources. Heuristics are used to select the most informative components and condense them for a concise plot. The output is a flexible Scalable Vector Graphic (SVG) diagram that can be displayed in a web page or graphic illustration tool.

**Conclusion:**

The Lollipops software creates information-dense, publication-quality mutation plots for automated pipelines and high-throughput workflows in precision medicine. The automatic data integration enables clinical data security, and visualization heuristics concisely present knowledge with minimal user configuration.

## Background

Precision medicine is becoming more widespread, especially as a result of the decreasing costs of high-throughput next-gen sequencing (NGS) technology. The high levels of detail now available to clinicians make it possible to associate specific individual differences to more effective treatments [1]. With this high level of detail comes an increasingly large amount of data that is difficult to process and understand efficiently. In many of these problem domains, particularly oncogenomics, the turnaround time from sample collection to initiation of clinical treatments is paramount [2].

Automated pipelines are necessary to handle the large NGS data, sequence assembly, variant detection, and database integration and reporting necessary to distill raw data into actionable results. Spreadsheets and other tabular displays are difficult to scan through by eye, which can result in additional effort, omissions, and missed treatment opportunities [3]. The development of visualization techniques for high-throughput dissemination of individual results is important to help pathologists discover the most informative variants, especially in the clinical domain where there can be many sources of data and treatments with varying levels of effectiveness.

So-called “lollipop” diagrams, named for the circles-on-sticks representation when drawn, provide a clear and concise view of point locations in a genomic region. These plots have also been referred to as “needle plots” and “stem plots” although these are more general terms for similarly styled diagrams. The simplest lollipop diagrams began with methylation diagrams, in which (un)filled circles represent methylation state in various panel locations [4]. These diagrams succinctly show the differences in methylation over time or across population groups in a single visual presentation. Alternatively, the Pfam database uses lollipop markers to depict active site residues in a 1-dimensional protein domain plot, showing the important components and positions of a complex 3-D protein without visually complex or hard-to-understand graphics [5]. Most recently, the cancer resource cBioPortal has provided a Mutation Mapper tool that displays the prevalence of selected mutations within the database of cancer samples [6]. This tool clearly shows the most common or rare variants out of a large collection of data, but is not well-suited to annotating a single individual sample. Mutation mapper provides methods for diagram export and powerful tools for exploring the effects on 3D protein structure, but cannot easily be automated, extended, or incorporated into novel tools such as those necessary for precision medicine.

There are a few publicly available, easy-to-use, extensible software packages for generating lollipop plots suitable for potentially sensitive clinical data (Table 1). One example is Plot Protein, an R script for creating detailed protein conservation and mutation plots. Plot Protein can provide many levels of detail and supporting information but requires the user to manually fetch, format, and integrate data from multiple sources [7]. Tools such as Mutation Mapper at cBioPortal are oriented to presenting population-level statistics and not individual variants, leading to inappropriate diagram layouts. While these tools are useful for studying the functional characterization of proteins, the sample counts provide too much extraneous detail when concerned with a specific individual loss of function.

**Table 1:** Features of software similar to the Lollipops tool.

Name	y-axis	domain coords	scriptable	dependencies
Mutation Mapper [6]	sample count, req.	auto	no	internet
Lollipops (this)	none	auto	yes	internet
Pfam [5]	none	auto	yes	JS, internet
muts-needle-plot [8]	sample count, req.	manual	yes	JS
trackViewer / Lolliplot [9]	custom, opt.	manual	yes	R
Plot Protein [7]	none	manual	yes	R

Selected features: *y-axis* indicates optional y-axis values and labeling. *domain coords* indicates that domain coordinates must be provided by the user (versus automatically fetched). *scriptable* indicates the source code can be modified to integrate with existing tools and/or customized to fit report styles. *dependencies* indicates if the tool requires anything additional to function such as internet access, a JavaScript runtime environment (JS), or the R statitistical software package (R).

The ‘Lollipops’ software tool presented here was specifically developed to serve clinical applications in precision medicine, but is flexible enough to be applied broadly. It provides the community with a simple tool usable by both non-technical users and bioinformaticists alike. The tool requires as little input as possible so that users do not need to integrate multiple data sources themselves, and produces clear SVG (Scalable Vector Graphics) diagrams viewable in any modern web browser or graphic illustration tool. This tool requires no additional software to run and can be easily integrated into novel pipelines or reports.

## Implementation

The Lollipops software consists of three major concepts: the automatic data fetcher, the data highlighter, and the SVG presentation. These concepts are intertwined but allow the tool to be easy-to-use, easy-to-interpret, and easy-to-extend with interactive features, respectively.

The automatic data fetcher is an important part of the tool’s ease-of-use for non-technical users. It works in two phases: identifier translation and subsequent protein domain retrieval. First, it takes an HGNC Gene Symbol (the only required parameter), and queries the Uniprot REST API to return the matching Uniprot/SwissProt Accession [10]. It then uses the fetched accession to query the Pfam graphic domain REST API endpoint [5]. The Pfam response data contains useful annotations for curated Pfam-A domains, regions of interest (signal peptides, transmembrane domains, etc.), and structurally interesting predicted areas such as coiled-coils, disordered, and low-complexity regions. This information is extracted and passed to the data highlighter and presentation methods.

**Fig 1:**
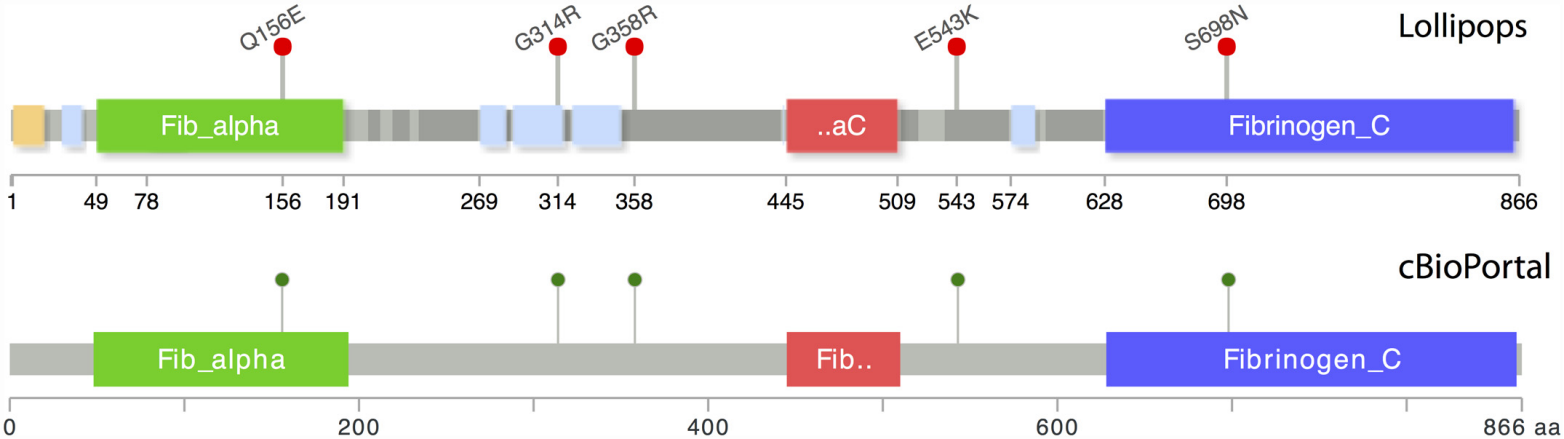
Comparison of FGA plots from Lollipops tool (top) and cBioPortal (bottom). Lollipops selects more informative axis labels and shows greater information density with the same plot size. Amino acid axis labels for domain start and stop positions, as well as exact marker locations, are clearly displayed for precision interpretation. In addition, lollipop labels are supported, and putative disordered regions (dark gray), low complexity regions (cyan), and signal peptides (orange) show additional structural information from Pfam without excessive detail.

The data highlighter encompasses straightforward techniques to make complex information easier to interpret visually. Many protein maps include an x-axis with regularly spaced amino acid intervals. This makes it difficult to visually estimate feature positions. The data highlighter prioritizes positions from the data to be presented and displays the most important positions for available space. For example, the tool assumes significant variants are presented in a table near the diagram (since supporting information and statistics are often necessary), but that protein domains are not (they are much more numerous and mostly unnecessary). Thus making sure the domain start/stop positions are displayed in the diagram ensures that the positions necessary to interpret the results are all readily accessible to the viewer—one can easily and accurately see the relative positions of the variant calls within a region of interest (See Fig 1). The data highlighter also works by staggering lollipop heights to make closely positioned mutations easier to distinguish. The Mutation Mapper at cBioPortal cannot stagger heights because the Y-axis denotes prevalence in cancer populations.

The data highlighter also helps interpretation using dynamic domain labeling as space is available. Some protein domains are large, allowing a full description to fit in the diagram instead of symbolic identifiers. Similarly, many domains are small, so an informative abbreviation or substring will fit better. For example, TP53 contains the 3 Pfam domains named “P53_TAD”, “P53”, and “P53_tetramer”—when displayed in Mutation Mapper, these are shown as just “P53..”, “P53”, and “P53..”, respectively—providing little discriminatory information on the domains. The data highlighter selects the labels “..TAD”, “P53 DNA-binding domain”, and “..tetramer”, respectively when given the same font size and diagram width as cBioPortal.

The SVG presentation also includes predicted Pfam regions of interest: low-complexity (light blue), signal peptides (orange), and disordered regions (darker grey). These annotations provide information about the possible effects of variants on protein structure in addition to active domains. These regions can be shown/hidden with a simple command-line option.

Finally, the SVG format vector diagrams allow for publication-quality graphics that scale well without pixelation artifacts. The readable, XML-based representation allows for editing and post-processing through automated pipelines or vector illustration software. All modern web browsers support embedding SVG into HTML pages, which enable automated workflows and interactive features such as tooltips and web links.

Although much of the implementation details are automated, certain pieces may be configured manually. Specific proteins may be specified by Uniprot/Swissprot ID, skipping the identifier translation step (with -U option). Lollipop colors may be individually specified by appending the variant with standard #RRGGBB color definitions. Lollipop scaling can be applied by appending the variant with e.g. 42 for a lollipop representing 42x the standard size (N.B. scales are relatively transformed to keep the plot informative). Finally, variant labels suitable for printing or publication can be added by specifying the -labels option (useful when supporting variant tables are not available). Each of these options, and others not mentioned, can be seen by running lollipops -help or found in the README.

## Results and Discussion

The plots in Fig 1 compare the same settings for lollipops and Mutation Mapper as applied to the *FGA fibrinogen alpha chain* subunit gene. First, the “Fib_aC” domain label is more informative given the space available, showing the alpha-C label (“aC”) instead of “Fib” prefix found in all 3 domains. The start and end locations for each of the 3 Pfam domains, and the exact locations of all 5 protein modifications are easily visible in the x-axis without reference to a separate table.

Other improvements show variant context and possible relations to protein activity. Variants that occur outside of active sites may be less interesting to clinicians who can focus on other findings presented. In Fig 1, we can see 2 variants at positions 314 and 543 which occur in disordered / low-complexity regions of FGA. These additional annotations do not overly complicate the diagram but provide more information about the implications of the variant calls.

This tool is best suited to single-sample variant analysis reporting. For cancer surveys, population-oriented tools are better suited to showing prevalence and important hotspot regions. For scientific discovery projects where more customization is needed, the scriptable interfaces found in R may be more suitable. The differences between tools can be seen in Table 1.

Because this tool is intended for use in a sequence variant pipeline, verification features and direct sequence access have not been implemented (they are typically present in earlier stages). These restrictions keep the code fast, light, and easy to integrate. The open source code base could be easily extended to incorporate additions. Future development could also extend this project into genomic sequence, as the prevalence of whole-genome sequencing expands.

## Conclusion

Precision medicine requires the ability to quickly pinpoint individual differences and estimate therapeutic effectiveness. Through the presentation of clear variant diagrams and relevant supporting information, tools such as lollipops allow clinicians to meet these needs head-on. Clear and concise visualization of individual sequence variants, overlaid on discernable protein diagrams, provide a valuable addition to automated reporting pipelines that need quick turnaround times.

The tool can be adopted for use with sensitive clinical datasets and internal projects, and further development can add missing features easily instead of starting from scratch. This tool is entirely written in publicly available Go source code and binaries available at https://github.com/pbnjay/lollipops with no required dependencies. This allows the software to be easily used in server environments, workstations, and laptops without issue.

Ensuring that information-dense, interpretable diagrams are used in future tools, publications, and data resources helps distill huge high-throughput next-gen data sets into powerfully useful and concise resources for clinical settings, and enables the next round of applications in precision medicine.

## Availability and Requirements

**Project name:** Lollipops

 **Project home page:** https://github.com/pbnjay/lollipops

 **Operating system(s):** Platform independent

 **Programming language:** Go

 **License:** GPL

 **Any restrictions to use by non-academics:** None
